# Artificial intelligence for precision malaria control: transforming surveillance, prediction, and intervention strategies

**DOI:** 10.3389/fdgth.2026.1879638

**Published:** 2026-08-06

**Authors:** Mohamed Sharif Abdi, Abdirahman Mohamed Adan, Naima Ibrahim Ahmed, Liibaan Abdulahi Sudi, Yusuf Hared Abdi, Mohamed Daud Mohamed, Yakub Burhan Abdullahi, Sharmake Gaiye Bashir, Ilyas Abdullahi Khalif, Abdikadir Ahmed Hassan

**Affiliations:** 1 Faculty of Health Sciences and Tropical Medicine, Somali National University, Mogadishu, Somalia; 2 Center for Health Research and Innovation, Somali National University, Mogadishu, Somalia; 3 Faculty of Health Science, Salaam University, Mogadishu, Somalia

**Keywords:** artificial intelligence, machine learning, malaria surveillance, outbreak prediction, precision public health

## Abstract

Malaria elimination has stalled globally despite decades of investment, with traditional surveillance constrained by retrospective reporting and limited capacity to integrate high-dimensional, non-linear data. In this perspective, we argue that artificial intelligence (AI) encompassing machine learning and deep learning can help shift malaria control from reactive reporting toward predictive, precision public health, while cautioning that it is one enabler among many rather than a stand-alone solution. We examine AI across three interconnected domains: surveillance (understood broadly as case detection, entomological and intervention-coverage monitoring, and the data-to-response loop), prediction (outbreak forecasting and spatial risk mapping), and control (resource stratification and intervention optimisation). Reported AI diagnostics can exceed 90% accuracy, and forecasting models offer useful lead times by integrating climatic, entomological and epidemiological data. However, realising this potential depends on resolving data-quality limitations, algorithmic bias, weak digital infrastructure, and absent governance frameworks, and on locally adapted rather than uniformly generalised models. We contend that demand-driven integration into national strategies, local capacity building, and prospective trials not algorithmic novelty will determine whether AI meaningfully accelerates progress toward elimination.

## 1 Introduction

Despite decades of investment, progress against malaria has stalled, with reductions in morbidity and mortality plateauing in recent years (1). Global malaria deaths had in fact declined steadily through the early 2000s from roughly 864,000 in 2000 to about 576,000 in 2019 before this progress reversed following COVID-19-related service disruptions and a convergence of biological and systemic threats (2). By 2022 the World Health Organization (WHO) estimated 249 million cases and 608,000 deaths, leaving the burden well above pre-pandemic levels; the most recent figures (an estimated 263 million cases and 597,000 deaths in 2023) confirm that case incidence has plateaued even as mortality has begun a slow post-pandemic decline (2). This burden remains overwhelmingly concentrated in the WHO African Region, which accounts for approximately 94% of cases (2).

The “High Burden to High Impact” (HBHI) countries, intended to drive global reductions, have instead seen case counts rise under a convergence of biological threats insecticide and antimalarial-drug resistance, Pfhrp2/3 deletions, and the invasive vector Anopheles stephensi and structural challenges more complex than technical factors alone. These include socioeconomic inequality, poverty and unequal access to care, fragile health systems, conflict-driven displacement, rapid population growth, climate change, widening funding gaps, and COVID-19-related disruptions (2–4). This deadlock suggests that current strategies, although essential, are insufficient to reach the 2030 elimination targets without innovation in how interventions are targeted and deployed (5, 6).

A primary driver of this stagnation is the limitation of traditional surveillance and response systems, which often rely on passive, retrospective data collection (7, 8). Importantly, malaria surveillance is not merely diagnosis: it encompasses case detection, entomological and vector monitoring, tracking of intervention coverage, and the data-to-response feedback loop that allows programmes to act on what is detected (“surveillance as an intervention”). In practice, passive systems aggregate facility data through processes plagued by reporting delays, incompleteness, and poor spatial resolution (8, 9), functioning as historical registries rather than early-warning mechanisms and rendering national malaria control programmes (NMCPs) reactive (10, 11). By the time an upsurge is detected through routine Health Management Information Systems (HMIS), transmission may already have peaked (12). Traditional statistical methods also struggle to integrate the high-dimensional, non-linear sources satellite remote sensing, genomic sequencing, and mobility data needed to capture malaria's ecological complexity (13).

Artificial Intelligence (AI), encompassing machine learning (ML) and deep learning (DL), has emerged to address these analytical bottlenecks (14). Unlike conventional statistics, AI can identify complex patterns within massive, heterogeneous datasets, enabling a shift from descriptive reporting to predictive intelligence (14). Applications range from computer-vision algorithms that automate microscopy to models that forecast outbreaks weeks ahead from hydroclimatic variables (15). Yet the literature remains fragmented and pilot-focused rather than systemically integrated (14). In this review, we examine AI across three interconnected domains surveillance, prediction, and control and argue that moving beyond fragmented, short-lived pilot projects (so-called “pilotitis”) toward sustainable implementation embedded within, and strengthening, national health systems is the central challenge.

## 2 Materials and methods

### 2.1 Study design

This study was conducted as a structured narrative review aimed at critically synthesizing current evidence on the application of artificial intelligence (AI) in malaria surveillance, prediction, diagnosis, vector control, and precision public health. A narrative review approach was selected because the objective was to integrate multidisciplinary evidence spanning epidemiology, artificial intelligence, digital health, health informatics, implementation science, and health-system governance rather than quantitatively synthesize intervention effects. In addition to summarizing existing evidence, the review adopted an analytical and comparative perspective, linking AI model development with operational implementation, governance, equity, and health-system strengthening.

### 2.2 Literature search strategy

A structured literature search was conducted across major scientific databases, including PubMed/MEDLINE, Scopus, Web of Science, IEEE Xplore, and Google Scholar, as shown in Table 1. The search covered publications from January 2015 to June 2026. This timeframe was selected to capture the rapid evolution of artificial intelligence technologies alongside recent advances in malaria surveillance, diagnosis, prediction, vector control, and precision public health. A limited number of earlier landmark publications were included when necessary to provide historical context and describe foundational AI concepts.

**Table 1:** Databases and search strategy.

Database	Search strategy (example Boolean query)
PubMed/MEDLINE	(“artificial intelligence” OR “machine learning” OR “deep learning”) AND (“malaria” OR “malaria surveillance” OR “malaria diagnosis” OR “malaria prediction” OR “malaria forecasting”) AND (“precision public health” OR “digital health” OR “vector control”)
Scopus	TITLE-ABS-KEY (“artificial intelligence” OR “machine learning” OR “deep learning”) AND TITLE-ABS-KEY (“malaria” OR “malaria surveillance” OR “malaria diagnosis” OR “malaria prediction”) AND TITLE-ABS-KEY (“digital health” OR “precision public health” OR “vector control”)
Web of Science	TS = (“artificial intelligence” OR “machine learning” OR “deep learning”) AND TS = (“malaria” OR “malaria surveillance” OR “malaria diagnosis” OR “malaria prediction”) AND TS = (“precision public health” OR “digital health” OR “vector control”)
IEEE Xplore	(“artificial intelligence” OR “machine learning” OR “deep learning”) AND (“malaria” OR “malaria diagnosis” OR “malaria prediction”) AND (“digital health” OR “decision support”)
Google Scholar	(“artificial intelligence” AND “malaria”) AND (“malaria surveillance” OR “malaria diagnosis” OR “malaria prediction” OR “vector control” OR “precision public health”)

AI, artificial intelligence; WHO, world health organization.

Search terms were developed iteratively and combined using Boolean operators. Core concepts included “artificial intelligence,” “machine learning,” “deep learning,” “malaria,” “malaria surveillance,” “malaria diagnosis,” “malaria prediction,” “malaria forecasting,” “malaria control,” “vector control,” “precision public health,” and “digital health.” Additional relevant studies were identified through manual screening of the reference lists of selected articles and key reports from international organizations, including the World Health Organization (WHO).

### 2.3 Eligibility criteria

Studies were selected based on their relevance to the objectives of this review. Inclusion criteria were: (1) publications addressing artificial intelligence, machine learning, deep learning, or related computational approaches in malaria surveillance, diagnosis, prediction, forecasting, vector control, decision support, or precision public health; (2) studies evaluating methodological, implementation, governance, or health-system aspects of AI in malaria control; (3) peer-reviewed original articles, systematic reviews, meta-analyses, implementation studies, and reports from international organizations; and (4) publications in English published between January 2015 and June 2026.

Exclusion criteria included studies unrelated to malaria, publications without sufficient methodological description, conference abstracts lacking full reports, and studies focused exclusively on non-healthcare AI applications unless cited solely as methodological analogies relevant to AI model development or validation.

### 2.4 Study selection and evidence synthesis

The literature selection process followed a structured narrative evidence-selection approach. Titles and abstracts were initially screened for relevance, followed by full-text assessment of potentially eligible publications. Because the objective was to synthesize multidisciplinary evidence rather than estimate pooled intervention effects, findings were analyzed thematically rather than quantitatively.

Evidence was organized into major domains relevant to AI-enabled malaria control, including (1) data foundations for AI systems, (2) AI-enhanced surveillance, (3) outbreak prediction and forecasting, (4) diagnostic applications, (5) decision support and intervention optimization, (6) implementation challenges, and (7) ethics, governance, and health-system readiness.

### 2.5 Quality appraisal and evidence interpretation

Because this study is a structured narrative review rather than a systematic review, a formal risk-of-bias assessment was not performed. Instead, evidence was critically interpreted according to methodological rigor, external validation, study design, relevance to malaria control, and applicability to real-world public health practice.

Particular attention was given to distinguishing algorithmic performance from operational impact. Studies reporting AI performance were interpreted alongside information on validation strategy, sensitivity, specificity, area under the receiver operating characteristic curve (AUROC), calibration, generalizability, implementation context, and reported limitations where available. Greater weight was given to malaria-specific AI studies, implementation research, and evidence derived from real-world public health settings.

### 2.6 Analytical framework

To improve analytical consistency, findings were interpreted using a conceptual framework linking AI development with malaria surveillance, prediction, implementation, governance, and health-system strengthening. This framework enabled comparison between technical model performance and real-world implementation readiness while identifying evidence gaps, methodological limitations, and future research priorities.

The framework also guided the interpretation of whether reported AI applications had demonstrated retrospective predictive performance, simulation-based benefit, pilot feasibility, or prospective public-health impact, thereby avoiding the assumption that high model accuracy automatically translates into improved malaria control outcomes.

## 3 Conceptual framework: the AI-enhanced malaria control ecosystem

Integrating AI into malaria elimination requires a clear view of the data-to-decision pathway and the points where computation adds value (16). Conventionally, microscopy, rapid diagnostic tests, and entomological surveys feed district and national databases such as DHIS2 (5), which are analysed descriptively to produce retrospective reports for annual planning. In this analog-to-semi-digital ecosystem the feedback loop is measured in months, creating a mismatch between transmission dynamics and intervention timing.

**Figure 1:**
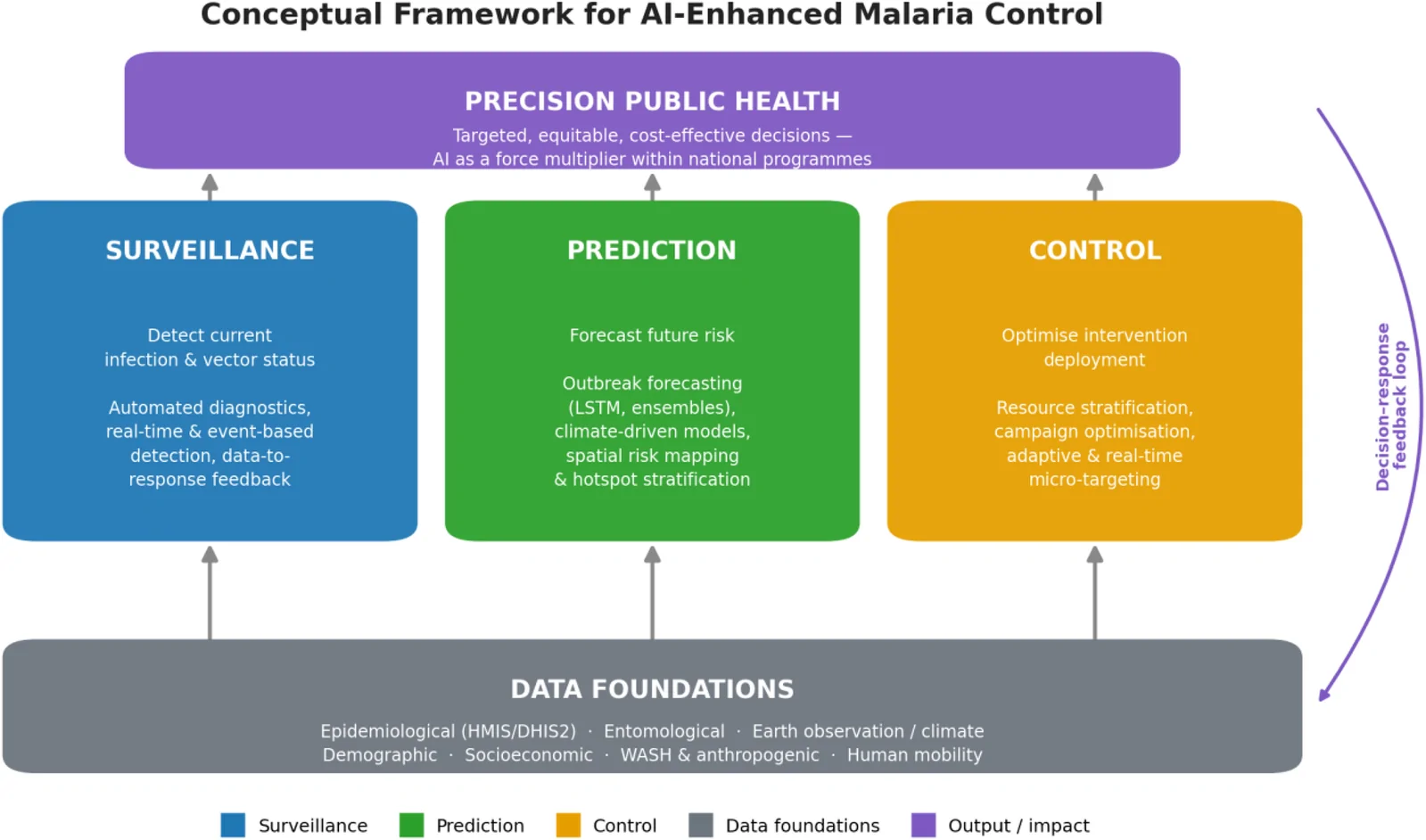
Conceptual framework for AI-enhanced precision public health in malaria control.

AI does not replace this pathway but accelerates and enriches it. In surveillance, convolutional neural networks (CNNs) can standardise parasite counting (15) and natural-language processing can mine non-traditional signals for real-time anomaly detection (13). In prediction, ML integrates surveillance data with environmental covariates to convert retrospective data into prospective intelligence (17). In control, optimisation algorithms translate risk maps into resource-allocation recommendations for example, for indoor residual spraying (IRS) or seasonal malaria chemoprevention (SMC) (18). Throughout, we distinguish AI as an analytic tool (“what is happening, or will happen?”) from AI as decision support (“what should we do?”), so that algorithmic accuracy is not conflated with operational utility (Figure 1).

## 4 Data foundations for AI-driven malaria systems

Robust AI for malaria is fundamentally constrained by data quality, granularity, and representativeness (19). Deep-learning models in particular require high-dimensional, disaggregated data to learn non-linear relationships (20). The current landscape shows a “rich–poor” divide: satellite-derived environmental data are abundant and continuous, whereas high-quality epidemiological and entomological data remain spatially fragmented and temporally sparse (19, 20).

Routine HMIS such as DHIS2 are the primary source of epidemiological training data (21), offering continuous longitudinal records but carrying substantial noise and bias (22). The completeness and structure of reporting vary substantially across countries and administrative units; in many settings though not all private-sector and informal-provider data are incompletely captured, creating blind spots that can bias predictions, an effect often most pronounced in urban areas where private-care utilisation is high (22–24). Because passive surveillance reflects health-seeking behaviour, asymptomatic infections and remote populations are systematically under-represented, leading models to underestimate transmission in the most vulnerable communities (24).

To compensate, models rely on Earth-observation covariates to predict habitat suitability and transmission risk (19). Sentinel-2 and Landsat support vegetation indices, land-surface temperature, and water-body detection, but a key limitation is spatial-resolution mismatch: precipitation products such as CHIRPS (∼5.5 km) may be too coarse for the micro-hydrology of small Anopheles breeding sites, and optical sensors are frequently obstructed by tropical cloud cover, producing gaps that require imputation before time-series forecasting (19).

Entomological data represent the most critical gap. Unlike routinely collected epidemiological data, vector surveillance is typically sporadic and project-based (25), data on density, species composition, and insecticide resistance are heterogeneous and poorly standardised, hampering generalisable models and forcing reliance on environmental proxies that introduce uncertainty when ecological associations break down (25).

Three recurrent data pathologies illustrate how “garbage in” becomes “garbage out” (26). First, class imbalance particularly in microscopy datasets where negative slides vastly outnumber positives can lead computer-vision models to favour specificity over sensitivity (26). Second, limited geographic diversity means a model trained on Southeast-Asian images (e.g., P. vivax) may fail to generalise to African contexts (P. falciparum) owing to differences in species and smear-preparation protocols (20). Third, missingness in routine data is rarely random; it correlates with health-system weakness, so models may perform worst precisely in the districts that most need support (23). In each case a specific input defect produces a predictable algorithmic failure, making data quality a prerequisite not an afterthought for reliable, ethical AI decision support (26) (Table 2).

**Table 2:** Data sources used in AI-based malaria surveillance, prediction, and control.

Data Type	Typical Sources	Spatial Resolution	Temporal Resolution	Key Limitations for AI Modelling	References
Routine epidemiological	National HMIS (e.g., DHIS2), IDSR reports	Facility/district	Monthly–weekly	Reporting delays and under-reporting; exclusion of private sector; confounding by health-seeking behaviour.	(22–24)
Survey & prevalence	DHS, Malaria Indicator Surveys (MIS)	Cluster (GPS)/household	Every 3–5 years	Low temporal frequency prevents real-time detection; data quickly outdated.	(21)
Remote sensing (climatic)	CHIRPS (rainfall), ERA5 (temperature), MODIS (LST)	1–30 km (variable)	Daily–monthly	Cloud-cover obstruction in tropics; resolution often too coarse for micro-habitat modelling.	(19)
Remote sensing (landscape)	Sentinel-2, Landsat 8/9, OpenStreetMap	10–30 m	5–16 days (revisit)	High processing cost; difficulty distinguishing breeding water bodies under canopy.	(19)
Entomological	VectorBase, localized field studies, IR Mapper	Point (sites)	Irregular/seasonal	Extreme sparsity; non-standardized collection; bias toward accessible sampling sites.	(25)
Diagnostic imaging	Microscopy slide banks, research datasets	Slide/cell	Cross-sectional	Class imbalance (few positives); staining/equipment variation limits generalizability.	(20, 26)
Demographic/population	WorldPop, national census, gridded population	Grid (≤100 m–1 km)	Census cycle/modelled	Projection error between censuses; undercount of mobile and displaced populations.	(27)
Socioeconomic	DHS wealth index, poverty surfaces, housing quality	Cluster/admin unit	Every 3–5 years	Low temporal frequency; socioeconomic inequalities strongly modify malaria exposure and healthcare access but remain underrepresented in many predictive models.	(28)
WASH & anthropogenic	Irrigation, land-use change, urbanization, sanitation coverage	Variable	Irregular	Heterogeneous data sources; anthropogenic environmental modifications such as irrigation, urban expansion, deforestation, and sanitation infrastructure substantially influence breeding-site formation and malaria transmission.	(29)
Human mobility	Mobile call detail records (CDRs), mobility datasets	Aggregated cells	Near real-time	Privacy and access constraints; mobility patterns vary during conflict, displacement, seasonal migration, and urbanization, reducing prediction accuracy when omitted.	(30)

## 5 AI-based malaria prediction and forecasting

The shift from reactive surveillance to proactive anticipation is among AI's most significant contributions to malaria control (31). Long Short-Term Memory (LSTM) networks and hybrid ensemble models (e.g., XGBoost–LSTM) have demonstrated promising performance in short-term malaria forecasting using historical datasets from endemic settings such as The Gambia and Bangladesh (31–34). Using historical datasets from endemic regions such as The Gambia and Bangladesh, LSTM and hybrid ensemble models (e.g., XGBoost–LSTM) have frequently outperformed traditional autoregressive models (ARIMA) in short-term malaria forecasting. However, reported performance varies substantially depending on the study population, prediction horizon, outcome definition, and validation strategy. Performance should therefore be interpreted using multiple metrics, including sensitivity, specificity, area under the receiver operating characteristic curve (AUROC), area under the precision–recall curve (AUPRC), calibration, and external validation, rather than accuracy alone, particularly in imbalanced surveillance datasets (31). Importantly, strong predictive performance does not necessarily translate into improved public-health outcomes (35). Most published AI studies report retrospective model performance or simulation results, whereas relatively few have evaluated whether AI-supported decision-making reduces malaria incidence, mortality, or operational costs under routine program conditions (36–38). Future studies should therefore prioritize prospective implementation research and pragmatic evaluations that assess epidemiological impact alongside predictive accuracy (35, 37).

Climate-informed AI models extend this foresight by integrating environmental variables together with anthropogenic and social determinants that influence mosquito ecology, human exposure, healthcare access, and parasite transmission (33). Malaria transmission is intrinsically linked to hydroclimatic variables, and temperature in particular exerts a strong, non-linear influence: beyond determining the duration of the parasite's sporogonic cycle, it shapes larval development, adult mosquito survival and longevity, biting frequency, and vector competence, with transmission peaking around the low-to-mid 20s °C and declining sharply at higher temperatures (33). Rainfall and humidity, in turn, regulate the availability and persistence of breeding sites (33). Random Forest, gradient boosting, and deep-learning models increasingly integrate climatic variables together with demographic, socioeconomic, human mobility, land-use, and health-system indicators to forecast malaria risk more accurately than climate-only approaches, reflecting the multifactorial nature of malaria transmission. In addition, models incorporating satellite-derived precipitation, land-surface temperature, and other environmental covariates have successfully forecast vector abundance and malaria incidence with lead times of 1–2 months, providing valuable early-warning capacity in epidemic-prone, climate-sensitive settings such as during El Niño events (33).

Although climate variables remain fundamental predictors of malaria transmission, increasing evidence indicates that incorporating anthropogenic and social determinants substantially improves predictive performance (28–30). Human mobility, population density, urbanization, irrigation schemes, land-use change, housing quality, poverty, sanitation, healthcare accessibility, and conflict-driven population displacement all influence human-vector contact, parasite importation, and local transmission dynamics (28–30). Machine-learning models that integrate these variables alongside climatic, epidemiological, and entomological data can better capture the complex ecological and socioeconomic drivers of malaria risk than climate-only models (19, 33). Such multidimensional modelling is particularly important in rapidly urbanizing settings and humanitarian contexts, where transmission patterns are increasingly shaped by human activities rather than environmental conditions alone (23, 35).

Beyond temporal forecasting, CNNs and spatial deep learning process high-resolution imagery to identify micro-ecological features linked to vector habitats, generating granular risk maps that stratify areas to village or household level (18, 39). This microstratification enables precision public health, targeting interventions to high-risk foci rather than applying them uniformly (18).

The operational utility of predictive models depends on an honest assessment of their transferability (34). A recurring limitation is overfitting to local context, so a model trained on P. falciparum dynamics in humid West Africa may fail for P. vivax in the arid Horn of Africa without recalibration (40). Validation frameworks such as “leave-one-region-out” cross-validation are valuable not because AI should yield a single universal model malaria's ecological and social heterogeneity makes locally adapted approaches essential but because they reveal how far performance degrades when a model is moved to a new setting. The practical goal is therefore transferable, locally recalibrated models retrained on local data, complemented by explainable-AI (XAI) techniques so that policymakers can trust why an outbreak is predicted (34, 40). Future malaria prediction frameworks should therefore move beyond predominantly climate-based modelling toward integrated socioecological approaches that jointly consider environmental, epidemiological, anthropogenic, and social determinants of transmission.

## 6 AI-informed malaria control: decision-making and action

Malaria interventions have historically followed “one-size-fits-all” policies that distribute scarce resources inefficiently (41). AI enables precision control by tailoring strategies to local epidemiology (42). In vector control, decision-support systems use stratification algorithms to optimise IRS and long-lasting insecticidal nets (LLINs) (43). Crucially, vector-control failure is rarely attributable to a single cause: it reflects interacting factors including insecticide resistance, shifts in vector behaviour (e.g., outdoor and early-evening biting), net durability and physical integrity, and the social and behavioural determinants of consistent net use. Within this complex picture, AI can address one tractable component spatial targeting by integrating entomological resistance data with transmission models to identify clusters where standard pyrethroid nets are failing and to recommend next-generation nets (e.g., PBO or dual-active-ingredient nets) (42). Given the high cost of these tools, simulation studies suggest AI-guided prioritisation can meaningfully increase campaign impact relative to uniform distribution, helping premium commodities reach areas of highest resistance intensity (43).

AI also supports resource allocation and prioritization (44). Optimisation methods, including reinforcement learning, weigh budget constraints, feasibility, and predicted outcomes to recommend an “optimal policy mix” for example, whether marginal funds are better spent expanding SMC coverage or IRS frequency (43). By simulating many scenarios, these tools provide NMCPs with evidence-based rankings, an asset amid shrinking donor funding (43, 44).

Finally, AI enables adaptive, near-real-time response. In contrast to rigid multi-year campaign cycles, AI-enabled systems can ingest live surveillance to trigger micro-campaigns for example, mobilising community health workers for focal mass drug administration before a localised outbreak spreads (45). Comparable logic extends to commodity security, where supply-chain forecasting predicts stock-out risk for RDTs and ACTs (45), and to community engagement, where behavioural-insight models tailor messaging to local barriers to uptake (46, 47). Realising these gains, however, requires robust supply chains and continuous, high-quality data streams (Table 3).

**Table 3:** AI applications for malaria control and programmatic decision-making.

AI Output Type	Malaria Control Application	Decision Supported	Operational Benefit	Implementation Challenges	References
Resource stratification maps	IRS & LLIN distribution	Prioritizing high-burden areas for targeted spraying and net allocation.	Directs limited commodities to areas of highest transmission intensity.	Requires high-resolution spatial data; granular maps may conflict with administrative boundaries.	(36)
Deployment optimization algorithms	Campaign planning & logistics	Optimal timing and routing for MDA or spraying teams.	Reduces cost and travel time; ensures coverage before peak transmission.	Needs accurate road/transport data, often lacking.	(36, 37)
Integrated resistance surveillance (phenotypic + genomic + entomological)	Drug & insecticide resistance management	Selecting insecticide classes and drug regimens, informed by bioassays, molecular markers, and vector behaviour — not genomics alone.	Pre-empts intervention failure by switching products before resistance becomes widespread.	Cost of sequencing/bioassays; sampling-to-insight lag; expertise gaps; behaviour not captured by markers.	(25, 46)
Supply-chain forecasting	Commodity security (RDTs, ACTs)	Predicting stock-out risk and optimizing inventory across facilities.	Prevents stock-outs of life-saving medicines; reduces wastage from expiry.	Poor LMIS data quality; reliance on historical consumption trends.	(45)
Behavioural-insight models	Community engagement & education	Tailoring messages to specific barriers to uptake (e.g., net use).	Increases adherence to and acceptance of interventions.	Requires qualitative social-science data; cultural nuance hard to model.	(46, 47)

## 7 Implementation, ethics, and governance

AI's potential is tempered by structural, ethical, and governance challenges (48). A primary bottleneck is digital readiness: many high-burden settings combine intermittent connectivity, paper-based reporting, and a limited technical workforce (48). Deployments of AI-driven tools such as automated RDT readers have exposed hardware failures and supply-chain delays, underscoring the risk of high-tech solutions in low-resource environments without commensurate system strengthening (38). Without reliable “analog” foundations power, data literacy, hardware maintenance AI risks becoming a costly, under-used asset (38).

Deployment also raises questions of algorithmic bias and equity. Models trained on historical data that are richer in urban centres than in high-burden rural areas may systematically under-predict risk in marginalised populations, producing “allocative harm” (49, 50). Limited locally representative training data such as diverse parasite morphologies across African regions can also degrade diagnostic accuracy when tools are transferred across borders, potentially widening inequities (35).

Governance and trust form a third pillar. The “black-box” nature of many models conflicts with the transparency NMCPs owe to populations and donors, who cannot justify high-stakes decisions such as withdrawing IRS from one district on opaque outputs (50). Regulatory frameworks for AI in global health remain sparse, creating liability uncertainty, while data-sovereignty concerns argue for “sovereign AI” frameworks ensuring local ownership of data and models (51). Critically, many pilots are supply-driven, built by external actors with limited end-user input, yielding tools that solve technical rather than operational problems (52). For impact, AI must be demand-driven and embedded in national malaria strategic plans, shifting from “tech-first” to “system-first” implementation that strengthens rather than bypasses national systems (53, 54).

The reliability of AI systems also depends on rigorous methodological evaluation. Model development should minimize data leakage, apply temporal and geographic validation, appropriately address missing data and class imbalance, and benchmark AI models against simpler statistical approaches where appropriate (35). Furthermore, reproducibility through transparent reporting and local recalibration before deployment are essential to ensure that models remain reliable across different epidemiological settings (35).

Looking ahead, priorities include integrating multi-source data mobility (call detail records), genomics, and hyperlocal climate into “digital-twin” platforms that visualise transmission dynamically, underpinned by interoperability standards such as HL7 FHIR (16, 55). Anomaly-detection methods are needed for pre-elimination settings, where sparse, clustered cases demand sensitive “last-mile” detection and reactive tools to guide active case detection (56, 57). Above all, the field must move from retrospective analyses to prospective, cluster-randomised trials testing hard epidemiological endpoints for instance, whether AI-guided IRS reduces incidence more than standard campaigns and invest in regional centres of excellence so that African data scientists can build, retrain, and audit models locally (16, 51, 58). All advances should align with WHO's digital-health and malaria strategies so that AI serves as a public good rather than widening the digital divide (16, 49).

## 8 Discussion

AI has emerged as a powerful analytical paradigm for malaria, reshaping how surveillance data become actionable insight by automating trend detection, forecasting risk, and optimising the allocation of limited resources (14, 15). Yet the capability to detect patterns or predict outbreaks, in the absence of demonstrated epidemiological impact and health-system integration, remains insufficient (38, 48). As in other areas of digital health, the transition from proof-of-concept to programmatic deployment depends less on algorithmic novelty than on responsible implementation that prioritises transparency, equity in data representation, and local ownership of digital infrastructure (35, 49–51).

Several challenges will determine whether this potential is realised. First, the predictive performance reported in retrospective studies must be confirmed prospectively; the field needs cluster-randomised trials testing hard epidemiological endpoints rather than algorithmic accuracy alone (16, 58). Second, the “black-box” nature of many models conflicts with the accountability that national programmes owe to their populations and donors, underscoring the need for explainable AI together with clear governance and liability frameworks (50). Third, models must be locally recalibrated rather than uniformly generalized, because malaria transmission is jointly determined by ecological, climatic, anthropogenic, and socioeconomic processes, requiring AI models that integrate multidimensional predictors rather than relying predominantly on climatic variables (34, 40). Finally, without strengthening the “analog” foundations of power, connectivity, data quality, and workforce, even accurate tools risk becoming costly, under-used investments (38).

In our view, Future advances in AI-driven malaria prediction should emphasize integrated socioecological modelling, combining climatic information with anthropogenic and social determinants to improve model robustness, transferability, and operational relevance across diverse epidemiological settings. AI is therefore best understood not as a solution to malaria but as a force multiplier one component of a broader effort whose success still depends on sustained financing, effective vector control and chemoprevention, resilient health systems, and the social and political conditions for elimination (42, 44, 53). Realized responsibly and embedded in national malaria strategic plans, AI can help convert stalled progress into more efficient, better-targeted, and more equitable action (53, 54), it risks diverting scarce resources and widening the digital divide (49). Whether it accelerates progress toward elimination will hinge far more on implementation, governance, and equity than on algorithmic performance alone.
